# Using behavioral economics to promote advanced directives for end of life care: a national study on message framing

**DOI:** 10.1080/21642850.2020.1823227

**Published:** 2020-10-27

**Authors:** Christy Spivey, Tara L. Brown, Maureen R. Courtney

**Affiliations:** a Arlington College of Business, University of Texas, Arlington, TX, USA; b College of Nursing and Health Innovation, University of Texas at Arlington, Arlington, TX, USA

**Keywords:** Advanced directives, message framing, behavioral economics, end of life, social norm framing

## Abstract

Advance directives (AD) are a crucial method for individuals to communicate their directions regarding medical decisions to their families and health care professionals when they are no longer able to make these decisions for themselves. However, not many individuals have an AD. We present the results of a survey-based experiment on how message framing (positive, negative and social norm) in educational videos affects (a) the individual’s decision to acquire more information about an AD and (b) the change in stated likelihood of obtaining an AD. Our message framing is centered on the family burden aspect of end-of-life care. We also survey participants about which type of framing they view as more persuasive in terms of obtaining an AD. We find that participants who watched the negative framed video were more likely to request more information about ADs. However, for those who had not sought information on ADs prior to the study, positive framing has a small positive impact on the approximate change in stated likelihood of obtaining an AD. On average, positive framing is perceived as more convincing to obtain an AD. Ranking the positive framed video as first or second in terms of convincingness is correlated with self-reported creation of an AD, whereas ranking the negative framed video as first or second is correlated with not creating an AD.

## Introduction

### Background and rationale

Individuals make many decisions about their personal health care. How individuals make these decisions and influencing factors are critical to understand. Often health care decisions are complex, especially about end-of-life care. Applying behavioral economics techniques, such as framing effects and social norms, can assist researchers and clinicians to investigate and better understand individual decision-making to support desired health outcomes.

A critical but difficult task in health care is to ensure that end-of-life care is consistent with patient preferences. Many Americans will need critical healthcare decisions made near the end-of-life but will lack the capacity to make those decisions themselves (Silveira, Kim, & Langa, 2010). The document to communicate preferences regarding end-of-life medical decisions to family and health care professionals is called an advance directive (AD). ADs can be used by individuals of all ages, although older adults are more often encouraged to prepare an AD (Maller, 2013).

A systematic review of 150 US studies including almost 800,000 participants revealed that only 37% had completed an AD (Yadav et al., 2017). Of note, this percentage did not change significantly between the years 2011–2015 in the studies reviewed. Similar proportions of healthy adults and those with chronic diseases did not vary by AD completion percentage. Unfortunately, many years of research and legislative initiatives have done little to increase the use of ADs.

The current low percentage of AD completion among the general population supports the need for researchers to develop tailored strategies to increase this percentage (Moorman & Inoue, 2013; Pollack, Morhaim, & Williams, 2010). This research is needed to illuminate promising approaches to achieve higher rates of AD completion in this country. Behavioral economics with its focus on how individuals make important decisions may provide an important structure to explore new AD promotion strategies.

To our knowledge, no studies have investigated how individuals learn about what an AD is and why it is important. However, a search of the topic reveals multiple websites, lay organizations, and professional organizations focused on this topic. Many electronic health records (EHR) used in medical offices have a query if the patient has a current AD. It is unclear what, if any, follow-up is done if the patient answers no (Goddard & Courtney, 2017). Future studies should focus on what the best online and healthcare educational efforts might be to promote public awareness of and subsequent completion of ADs.

The benefits of having an AD accrue not only to the individual, but also to their family, their doctors, and society as a whole. ADs give individuals autonomy and peace of mind regarding healthcare decisions, avoiding an emotional burden on family that can take a toll for years to come. Also, doctors can honor patient wishes and values, which often results in a reduction in unnecessary tests and treatments. According to a GAO (United States Government Accountability Office) report in April of 2015, even though major healthcare institutions must have written policies about advance directives and their use, staff and patients remain challenged to discuss end-of-life care. This further affirms the need for strategies to achieve completion of ADs.

Unfortunately, even though many people would prefer to die more peacefully at home, over 80% of deaths occur in hospitals or nursing homes and are often associated with painful, expensive high-tech treatment (Bailey & Periyakoil, 2020; Steinhauser, Clipp, et al., 2000). With an AD in place, individuals might experience a more preferred and less aggressive end-of-life care process. Higher healthcare costs tend to be associated with care in the last few months of life (Riley & Lubitz, 2010; Unroe et al., 2011). Policymakers believe that major cost savings might be achieved with an increased use of ADs. Of note, Rao, Anderson, Lin, and Laux (2014, p. 2) reported that ‘advance directives were associated with significantly lower levels of Medicare spending, a lower likelihood of in-hospital deaths, and increased hospice use in regions characterized by higher levels of end-of-life (EOL) spending.’

Top reasons for not having an advance directive (AD) in a recent national study were not being aware of what an AD is and believing that family members already knew what the individual’s wishes were (Rao et al., 2014). Racial and educational disparities existed in AD completion with higher completion percentages for those who were white, older, and who had higher education and incomes (Rao et al., 2014). Higher completion percentages were also associated with having a chronic disease and a usual source of medical care.

Research suggests that a major motivator to establish an AD, even more of a motivator than personal autonomy, is to avoid burdening family members with difficult decisions at an emotionally-fraught time (Steinhauser, Christakis, et al., 2000). We propose to use this knowledge about human motivation and test how best to frame messaging about family burden to initiate action surrounding ADs.

Framing, a behavioral economics technique, posits that the way in which information is delivered matters to how individuals make choices. For example, the likelihood of an individual choosing to have a surgical procedure will differ depending on whether they are told the procedure has a 5% mortality rate (negative or loss-framed) or that it has a 95% survival rate (positive or gain-framed). Prior studies of framing related to health have shown that positive frames are more effective for prevention, while negative frames are more persuasive for detection. Rothman and Salovey (1997) found that loss-framed messages work better to promote detection procedures such as mammograms, while gain-framed messages seem to be more effective in promoting prevention behaviors, such as using sunscreen. Meyerowitz and Chaiken (1987) found that negative framing was associated with more positive intentions and behaviors around breast self-exam. Similarly, Thaler and Sunstein (2008, p. 159) pointed out that ‘people are more likely to engage in self-examinations for skin and breast cancer if they are told not about the reduced risk if they do so but about the increased risk if they fail to do so.’ Clarifying a strategy goal, such as promoting prevention or detection, is essential to creating an appropriate frame.

McNeil, Pauker, Soc, and Tversky (1982) studied the effects of framing and full information disclosure to patients with lung cancer. They found patients’ choices of surgery vs radiation therapy were affected by the framing of treatment possibilities in terms of life expectancy (positive frame) vs the probability of dying (negative frame), such that surgery was more attractive when the problem was framed in terms of the probability of living rather than in terms of the probability of dying. Ledgerwood and Boydstun (2014) investigated what happens when frames change over time: from negative to positive (i.e. loss-to-gain) and positive to negative (i.e. gain-to-loss). In their novel investigation of changing frames, they found that negative framed messages are sticky; in other words, it’s harder for individuals to switch from thinking about something framed negatively to something framed positively than vice versa. Our study also employs a within-subjects design and uses negative and positive framing, though the focus is not on the impact of changing frames over time. In addition, our study examines the impact of social norms on behavior.

Social norms describes the phenomena in human behavior in which people’s actions are influenced by what they perceive those around them are doing. There are several studies that explore the effect of social norms on health decisions and related issues. Zimmerman (2009) studied the use of social norms to promote physical activity. He found social norms is one of a couple anchors that motivate people’s decisions on the degree and duration of physical activity. He argued promotions using (among others) a social norm anchor of physical activity of others can motivate people to increase their own physical activity.

Akin to that, powerful stories about events that happen to others can be especially effective in motivating behavior rather than statistics, especially for those who are not quantitatively inclined. A person’s story has the power to affect human behavior more than ‘faceless statistics’ (Courtney, Spivey, & Daniel, 2014). An example of the effect of a person’s story is documented by Ubel, Jepson, and Baron (2001). In their study, participants received hypothetical statistical information about treatment success using angioplasty and bypass surgery for angina patients. They also received patient testimonials from successful and unsuccessful procedures of both types- some treatments conforming to the statistical success rate already given and some other treatments not conforming to the statistical treatments given. They concluded testimonials greatly influenced the hypothetical choice of procedures from participants. These patient testimonials provided the social norm framing used in this study. To our knowledge there are not any studies that directly study the social norm frame and its effect on actions taken around ADs.

There are a few studies that involve behavioral economics and advance directives. Halpern (2012) specified five cognitive biases that may affect the decision to obtain an advance directive. His study did not include investigation of positive or negative message framing. Halpern et al. (2013) also studied the importance of the type of end-of-life care a patient desires (i.e. comfort oriented or life extending oriented) and found most patients prefer comfort oriented care unless there is a default mode selected. Kressel and Chapman (2007) examined the default option and its effects on describing a patient’s true end-of-life care desires. While important, these studies are outside the scope of our study as we focus on how framing affects the decision to take action around ADs, not what choices are made in the enrollment process.

### Objectives

The intention of this study is to answer the following research questions:

1. How does the type of message framing, centered on the family burden aspect of end-of-life care, affect the likelihood of wanting to acquire more information about an AD and the change in stated likelihood of ever obtaining an AD?
2. Which type of message framing do participants perceive as most convincing to obtain an AD?
3. What factors are correlated with self-reported AD obtainment, speaking to someone about obtaining an AD, or stating an intention to obtain an AD?

The answer to these questions is difficult to predict given the existing literature. As suggested by various studies, loss-framed messages work better to encourage detection procedures, while gain-framed messages are more effective in promoting prevention behaviors. Choosing to get more information about an AD is not a detection procedure nor a prevention behavior, though we may view obtaining an AD as preventing against family angst in the future. Similarly, it’s not clear which type framing participants will view as more convincing in terms of taking actions beyond requesting more information about an AD. The negative framing may be more uncomfortable to watch, but if individuals are loss-averse it may be more motivating.

## Methods

### Study design and protocol

Following Steinhauser, Christakis, et al. (2000) we conducted an online survey-based experiment. We decided upon using a survey (as opposed to a clinical study such as Halpern et al. (2013) and other distinguished studies) for our particular study for several reasons. First, we wanted to ensure a diverse population to explore the effects of and attitudes toward framing for people of different ages and health conditions, as an AD could be needed at any moment due to unexpected occurrences. Second, we wanted to minimize any possibility of an experimenter effect. In a clinical study, participants could be more likely to say they wanted more information about an AD because of the pressure of being there in person. While some of this bias is mitigated through the use of electronic survey mechanisms (as opposed to an interviewer), just the difference in setting can have an impact. In our design, because of the removal of any face to face interaction, participants were likely more at ease to answer the questions as they best see fit. While we agree there is no perfect method with which to conduct research, for this particular study we found a survey to be the best fit. In addition, our study is somewhat exploratory in nature. It is mainly focused on the effect of the types of framing on wanting more information about an AD and the stated likelihood of creating an AD. The other research questions involve the perceptions of participants and factors that are correlated with taking action around ADs. It is possible the findings of this study will spur a larger study involving a clinical setting.

The survey was administered by Qualtrics.com, a survey development tool that also conducts surveys from their sample of recruited participants. Qualtrics samples comes primarily from research panels and online social media recruiting. Qualtrics uses Grand Mean certified sample partners to retain the integrity of the survey data by excluding duplication and ensure sample validity. Qualtrics checks every IP address and uses a sophisticated digital fingerprinting technology. Potential respondents are sent an email invitation informing them that the survey is for research purposes only, how long the survey is expected to take, and what incentives are available. To avoid self-selection bias, the survey invitation does not include specific details about the contents of the survey. Participants receive an incentive from Qualtrics. Qualtrics determines the incentive based on the length of the survey and target acquisition difficulty. The specific type of rewards vary and may include cash, airline miles, gift cards, redeemable points, sweepstakes entrance and vouchers (Qualtrics, 2014). Participants were randomly assigned identification numbers at project entry by the vendor. Qualtrics managed selecting participants and all communication with the participants. All eligible participants were given a written informed consent message approved by the University of Texas at Arlington IRB prior to participation that explained the study procedures and the subject matter.

Our study consisted of two parts. In part one, an initial survey, Qualtrics contacted a national sample of individuals, stratified by age. The sample consisted of 1160 adults aged 21–90, with equal numbers of participants from age groups 21–34, 35–49, 50–64, and 65 + . Most previous studies focused on ADs have focused on elderly and/or sick patients. However, we note that the need for an advance directive could occur at any stage in life due to car accidents or sudden illness. Hence, we see the importance of awareness and education of advance directives for all ages and health conditions.

The sample size of 1160 adults was decided a-priori to provide a large sample to support subgroup analyses using the Creative Research Systems calculator (2017). The confidence interval or margin of error for the overall sample size of >1000 was calculated as 3%. Specific power calculations were not conducted for this study.

In the initial survey, participants were first asked if they currently had an advance directive. If they answered yes, the survey ended. We do not have information on how many individuals exited the survey because they already have an AD, nor do we know any demographic characteristics of these individuals except that they were aged 21–90.

Participants who stated they did not have an advanced directive then saw an introductory one minute video describing what an advance directive is (Appendix A), which will be referred to as the informational video. They then were asked questions (Appendix B) related to their personal experience with ADs, such as whether they have ever sought information about ADs, whether they have ever spoken to family members about end-of-life care, and the reasons they currently do not have an AD.

Next, participants saw one of three videos with different message framing around the family burden aspect of end-of-life care.

- There is a 1 min and 28 s negatively framed video that features a brother, sister and sister’s husband in a hospital waiting room trying to make an end-of-life care decision for their father who is incapacitated and who did not have an advance directive. This family is in grief and worries over the decisions they should make given they don’t know their father’s wishes (we will refer to this video as negative framed video). See Appendix C for a transcript and link to this video.
- Another video is a 1 min and 7 s positively framed video that features the same family in the previous video. This time their father had an advance directive, so while in grief, they are relieved their father’s wishes are carried out (we will refer to this video as the positive framed video). See Appendix D for a transcript and link to this video.
- The final 1 min and 37 s video uses a social norm to frame the decision to make an advance directive.^1^ This video features two friends discussing a third friend’s experience when her husband was incapacitated but had no advance directive. The friends conclude an advance directive is a good thing and after doing research have decided to get one, too. See Appendix E for a transcript and link to this video.^2^

Video scripts were developed by the research team and reviewed by a clinical expert panel (5 nursing faculty experts). Videos were rated as to clarity and quality of message. Content validity was established at the 100% level. Additionally, a classroom pilot study of 35 students in a Master’s Economics class was conducted to obtain and analyze video ratings. Content validity was confirmed. The videos used professional actors and were approved by the UTA IRB.

Participants were randomly divided into three groups, which we will refer to as conditions. Individuals assigned to each condition saw the videos in a different order as described in Table 1.^3^

**Table 1.** Video Order by Condition.

Condition	First Video Viewed	Second Video Viewed	Third Video Viewed
1	Negative Framed	Positive Framed	Social Norm
2	Social Norm	Negative Framed	Positive Framed
3	Positive Framed	Social Norm	Negative Framed

In each condition, after participants viewed their first video (and were unaware of other videos coming), they were asked questions (Appendix F) about the video and their opinion of its effectiveness in motivating to obtain an AD. They were also asked if they would like more information on advance directives. If they indicated they would, they were given the opportunity to learn more about ADs and end-of-life planning through a link at the end of part one of the survey that directed them to the National Hospice and Palliative Care Organization’s website (www.caringinfo.org). They were also offered the opportunity to set up a free AD at mydirectives.com.

Participants then watched their second framed video. After the second video, participants were asked similar questions as they were about the first video they viewed plus some additional questions comparing the two videos (Appendix G).^4^ Participants then watched their third framed video. They were then asked the same questions as they saw after the second framed video plus questions comparing all three videos (Appendix H). After viewing all three videos, participants were asked to answer some demographic and viewpoint questions (Appendix I). As soon as they finished answering these questions, their participation in this stage was complete.

Part two of the study consisted of a brief follow-up survey conducted about one month after part one of the study, to determine if attitudes about ADs had changed and if participants had taken any self-reported actions surrounding ADs, including whether they had obtained an AD. Questions are available in Appendix J. For the follow-up survey, Qualtrics contacted all initial participants, and stopped the follow-up after 562 participants completed the follow-up study. The sample size of the follow-up survey was dictated by practicality (not all initial participants would respond to the follow-up) and budget.

Our design intentionally has a within and between participant design. In the initial survey, participants indicate their interest in receiving more information about an advance directive and their likelihood of creating an AD after only seeing one framed video. Hence, we are able to analyze which frame (positive, negative, or social norm), if any, affected these outcomes. Because of the random assignment to each condition, the relationship between framing type and these outcomes can be interpreted causally^5^, given the appropriate control variables. We then allowed the participants to see all of the videos and elicited their opinions comparing the videos as well as asking them to rank them in terms of which they perceived as most convincing in terms of obtaining an AD (the ‘within participant’ design).

In part two, the follow-up survey, we were able to see if the videos in the initial survey nudged participants to obtain an advance directive. Because participants had seen all videos at this point, we cannot interpret any causal relationship between type of framing and self-reported obtainment of an AD. This design, of course, limits our ability to distinguish which type of framing is the most effective in convincing participants to obtain an advance directive. We decided obtaining participants’ ranking of the videos was important to do in part one because of our uncertainty of return rates for part two of the survey. Since we are at the beginning of this type of research (using framing from videos to nudge individual’s behavior), we decided obtaining a ranking of how convincing the videos are in relation to each other was important even though we lost some control in determining exactly which video prompted action beyond wanting to learn more information. We plan to address this and other related questions in subsequent projects.

### Variables

Table 2 below defines important variables used in the empirical estimations.

**Table 2.** Variables of Interest.

Variable Name	Definition	Variable Type	When Measured
*SoughtInfo*	Dummy variable = 1 if individual has sought information about ADs prior to participating in this study	Control	Initial survey, after seeing informational video but before first framed video
*SeekLikelihood*	Indicates stated likelihood of ever seeking information about creating an AD. Ranging from Not at All Likely (1) to Very Likely (5).	Outcome	Initial survey, after seeing informational video but before first framed video; asked of those who indicated they had not previously sought info
*SpokenToFam*	Dummy Variable = 1 if individual has spoken to family members about wishes regarding end-of-life care	Control	Initial survey, after seeing informational video but before first framed video
*DecidedCare*	Dummy variable = 1 if individual has had to decide or help decide end-of-life care for someone else	Control	Initial survey
*ADlikelihood*	Indicates stated likelihood of obtaining an AD after viewing initial framed video. Ranging from ‘Not At All Likely’ (1) to ‘Very Likely’ (5).	Outcome	Initial survey, after seeing first framed video
*WantInfo*	Dummy variable = 1 to if individual indicated they wanted more information about ADs after viewing initial framed video (including how to set up a free AD)	Outcome	Initial survey, after seeing first framed video
*Condition1*	Dummy variable = 1 if individual saw the negative framed video first (saw positive second and social norm third)	Independent variable of interest	Initial survey
*Condition2*	Dummy variable = 1 if individual saw the social norm video first (saw negative second and positive third)	Independent variable of interest	Initial survey
*Condition 3*	Dummy variable = 1 if individual saw the positive framed video first (saw social norm second and negative third)	Independent variable of interest	Initial survey
*Rank (RankNeg, RankNorm, RankPos)*	Indicates perceived convincingness of videos; =1, 2, or 3, with 1 being most convincing	Outcome, Independent variable of interest	Initial survey, after seeing all videos
*CreatedAD*	Dummy variable = 1 if individual self-reported having created an AD about a month after the initial survey	Outcome	Follow-up survey
*SpokeAboutAD*	Dummy variable = 1 if individual self-reported they had spoken to someone about creating an AD within about a month of initial survey	Outcome	Follow-up survey; asked of those who had not created AD since initial survey
*WillCreateAD*	Dummy variable = 1 if individual self-reported they would create an AD soon or someday	Outcome	Follow-up survey; asked of those who had not created AD since initial survey

In addition to the variables of interest in the table above, we also consider a number of independent variables as controls. The continuous control variables include age and number of children. The categorical control variables include dummy variables for gender, relationship status (married, cohabitating, divorced, widowed, and never married), race (white, black or African American, Asian, American Indian or Alaskan native, other), Hispanic heritage, educational attainment (less than 9th grade, some high school, high school grad, some college, associate’s degree, bachelor’s degree, some graduate school, graduate degree), self-reported health status (excellent, very good, good, fair, poor), whether or not have a chronic health issue or disease, whether or not have health insurance, political views (conservative, moderate, liberal, other), frequency of church attendance (more than once a week, once a week, a couple of times a month, once a month, rarely, never), and reasons don't have an AD (see Table 4 below for various reasons). We also include variables measuring risk aversion, fatalism, free choice, and time preference, measured on a likert scale of 1–10. These variables are potentially correlated with decisions regarding ADs. For example, people who are more risk averse may be more likely to want to ensure end-of-life decisions are completed according to their wishes, which reduces uncertainty in the future. These are more fully explained in Table 3.

### Empirical models

In addition to descriptive statistics, we estimate several models to help answer the research questions. All models are estimated with robust standard errors.

Model 1: The first model we estimate is a probit model, to determine if a relationship exists between message framing and wanting to know more information about ADs after watching the first framed video.

Dependent Variable: *WantInfo*

Independent Variables of Interest: *Condition 1*, *Condition 2* (*Condition 3* is the base category)

Model 2: The second model is an OLS regression to determine if a relationship exists between message framing and a change in the stated likelihood of obtaining an AD. The initial likelihood is measured after watching the informational video, and the second stated likelihood is measured after watching the first framed video.

Dependent Variable: ADdiff = ADLikelihood - SeekLikelihood

Independent Variables of Interest: Condition 1, Condition 2 (Condition 3 is the base category)

Models 3a, b, c: The third model is an ordered probit for each video to determine what factors are correlated with perceived convincingness to obtain an AD. The ranking of perceived convincingness was measured after watching all three framed videos.

Dependent Variable: *Rank*

Models 4a, b, c: The fourth model is a probit to determine what factors are correlated with self-reported creation of an AD after the initial survey but before the follow-up survey. One of these factors is how participants ranked the videos in terms of convincingness. We also estimate probits for those who did not create an AD, to determine what is correlated with having reported speaking to someone about an AD and stating that an AD will be created someday or soon.

Dependent Variables: CreatedAD, SpokeAboutAD, WillCreateAD

Independent Variables of Interest: Dummy variables for ranking negative framed video as most convincing and ranking positive framed video as most convincing, with ranking social norm video as most convincing as base category (RankNeg = 1, RankNorm = 1, with RankPos = 1 as base category)

## Results

### Descriptive statistics

Table 3 presents demographic descriptive statistics for the sample in part one of the study (N = 1,160).

**Table 3.** Demographic Descriptive Statistics.

	Mean/Percentage
Age	47.57
Female	75.6%
Hispanic or Latino origin	9.7%
*Race:*	
White	79.4%
Black	13.4%
Asian	3.1%
American Indian or Alaskan native	0.78%
Other	3.4%
*Relationship Status:*	
Married	50.6%
Cohabitating	11.4%
Divorced	15.6%
Widowed	3.4%
Never Married	19%
Number of Children	1.66
*Educational Attainment:*	
No High School	.6%
Some High School	3%
High School Graduate	21.7%
Some College	29.6%
Associate’s Degree	12.8%
Bachelor’s Degree	20.9%
Some Graduate School	1.2%
Graduate Degree	10.3%
*Political Views:*	
Conservative	29.1%
Moderate	37.4%
Liberal	25.2%
Other	8.3%
*Religious Service Attendance:*	
More than Once a Week	7.6%
Once a Week	17.2%
A Couple of Times a Month	8.4%
Once a Month	4.5%
Rarely	38.2%
Never	24.2%
*Health Status:*	
Excellent	10.1%
Very Good	29.7%
Good	41.6%
Fair	15.8%
Poor	2.8%
Have a Chronic Disease	35.1%
Have Health Insurance	88.4%
Extent of freedom of choice and control you feel you have over the way your life turns out. Ranging from No Choice at All (1) to A Great Deal of Choice (10)	7.46
Extent believe that individuals can decide their own destiny, or it’s impossible to escape a predetermined fate. Ranging from Everything is Determined by Fate (1) to People Shape their Fate Themselves (10)	6.26
Generally a person who enjoys taking risks, or tries to avoid taking risks? Ranging from I avoid taking risks (1) to I enjoy taking risks (10)	4.73
Generally willing to sacrifice your immediate concerns to improve your future prospects, or more likely to satisfy your immediate concerns while reasoning that the future will take care of itself? Ranging from Sacrifice for the future (1) to Satisfy immediate concerns (10)	5.3

Demographic data validate a diverse sample across ages, race/ethnic, and education levels. It is noted that the high percentage of females may be influenced by the older age groups in the sample. Eighty percent of the sample rated their health as good to excellent. Only one-third of the sample reported a chronic disease. Overall, the sample may be healthier than expected and this may be related to individuals who choose to participate in Qualtrics studies. In general, participants tended to an internal locus of control. They were more mid-point regarding risk-taking preference and present vs future orientation.

Table 4 presents information on participants’ awareness of and history with advance directives and end-of-life care. These questions were answered before participants saw any framed video but after the informational video. About a quarter of participants had previously sought out some type of information about ADs, and over 50% of those who had not previously sought information said they were likely or very likely to ever seek out information moving forward. About half of participants had already spoken to family about their end-of-life wishes. Participants gave a number of reasons for not already having an AD, with the top two responses being that they keep meaning to get around to it and that no one has encouraged them to get one.

**Table 4.** Knowledge of and History with End-of-Life Care.

Question	Answers (N=1,160)
Have you ever sought information about an advance directive? (*SoughtInfo*)	Yes – 24.66% No – 72.41% Not sure- 2.93%
If no to above, how likely are you to ever seek information about creating an advance directive? Ranging from Not at All Likely (1) to Very Likely (5). (*SeekLikelihood*)	1- 7.89% 2- 8.58% 3- 31.01% 4- 29.75% 5- 22.77%
Have you spoken to your family members about your wishes for end-of-life care? (*SpokenToFam*)	Yes – 50.6% No – 46.8% Not sure – 2.6%
Why don’t you have an advance directive? Check all that apply. (*WhyNoAD1-WhyNoAD8*)	I didn’t know about them until now – 22% I have made my end-of-life decisions known to my family and that is enough – 17% I keep meaning to get around to it but have been delayed – 34.6% It makes me sad, uneasy, or uncomfortable to think about it- 23.5% The process seems too complicated and time consuming- 13.1% My doctor has never discussed an advance directive with me – 11.6% No one has encouraged me to get one – 27.2% 8. Other (most write in responses involved being young and/or not sick) 5.3%
Have you been in a situation where you had to decide or help decide about someone else’s end-of-life care? (*DecidedCare*)	No-61.2% Yes- 38.8% If yes, did that person have an advance directive? Yes- 31%; No- 62%; Not sure- 7%

### Part one of the study: between participants results

After seeing the first framed video, participants were asked the following two questions of immediate interest:

- How likely are you to ever obtain an advance directive, after seeing this video? Ranging from ‘Not At All Likely’ (1) to ‘Very Likely’ (5), please select one. (*ADlikelihood*)
- Would you like more information about advance directives, including how to set up a free advance directive? If so, we will provide that information at the end of the survey. (Yes/No) (*WantInfo*)

#### Descriptive statistics

Table 5 reports the results of how framing impacts the answers to these questions. There are small differences in the stated likelihood of obtaining an AD after watching the first framed video (*ADlikelihood*). The highest likelihood is associated with the social norm video, followed by the positive framed video and then the negative framed video. However, these differences are not statistically significant, according to a Bonferroni multiple-comparison test (F = 0.64, *p* = 0.525).

**Table 5.** Likelihood of Obtaining AD and Wanting More Information after Viewing First Framed Video.

Condition & First Video Observed	N	Mean of *ADlikelihood*	% of Yes Responses (*WantInfo*=1)	% of No Responses (*WantInfo*=0)
Overall	1,160	3.69	42.8	57.2
1- Negative Framed	377	3.64	49.1	50.9
2- Social Norm	388	3.73	42.0	57.2
3- Positive Framed	395	3.70	37.5	62.5

A higher percentage of the participants who viewed the negative framed video first indicated they wanted more information on ADs than any other video (*WantInfo*). There are statistically significant differences between the three condition groups in the proportion who chose to receive more information after viewing the first video, according to a Bonferroni multiple-comparison test (F = 5.41, *p* = 0.0046).

Recall that after the informational video, but before seeing the first framed video, participants were asked if they had ever sought information about ADs (*SoughtInfo*). Approximately 25% had done so prior to participating in our survey.

There is a relationship between wanting more information about creating an AD after viewing the first framed video (*WantInfo*) and whether a participant had sought information on ADs prior to our study (*SoughtInfo*). Of those who had sought information prior to our study (*n* = 286), 50% wanted more information about creating an AD after watching the first framed video, and 50% did not. Of those who had not sought information prior to our study (*n* = 874), 40% wanted more information after watching the first framed video, and 60% did not. So, those who had sought out information prior were 10 percentage points more likely than others to be information seekers after watching the first framed video.

There is also a relationship between stated likelihood of obtaining an AD after watching the first framed video (*ADlikelihood*) and whether a participant had sought information on ADs prior to our study (*SoughtInfo*). For those who had sought information prior to our study, the mean value of *ADlikelihood* is 3.95. For those who had not sought information prior to our study, the mean value of *ADlikelihood* is 3.6. Thus, the stated likelihood of obtaining an AD after watching the first framed video is higher for those who had already been information seekers.

Respondents who had not sought any information on ADs prior to the study (*n* = 874) were asked the following: How likely are you to ever seek information about creating an advance directive, on a scale of Not at All Likely (1) to Very Likely (5) (*SeekLikelihood*). We can use the question about likelihood of obtaining an AD after watching the first framed video (*ADLikelihood)* to assess a change in attitudes.

Table 6 below shows the averages of these variables of interest by condition for those who had not sought information about ADs prior to the study. As expected, we see no difference in the mean of *SeekLikelihood* across conditions according to a Bonferroni comparison test (F = .69, *p* = .5), since the assignment to groups was random and this question was asked prior to seeing any framed videos. We see slightly less variation in the mean of *ADlikelihood* across conditions, but these are not statistically significant (F = .53, *p* = .59). However, we do see variation in the difference of the means by condition, with the positive framed video having the largest increase in likelihood of obtaining an AD, followed by the social norm video. There was virtually no change associated with the negative framed video. This variation in the differences of the means is not statistically significant, according to a Bonferroni multiple comparison test (F = 1.93, *p* = .146). If we compare the difference in the means for two groups, those who saw the positive framed first and everyone else, the null hypothesis that the means are the same can be rejected at the 9.6% level (when the alternative hypothesis is that the means are not equal) and at the 4.8% level (when the alternative is that mean for condition 3 is greater than the means for all others).

**Table 6.** Likelihood of Seeking Information about Creating AD before Seeing First Framed Video vs. Likelihood of Obtaining AD after Viewing First Framed Video.

Condition & First Video Observed	N	Mean of *SeekLikelihood*	Mean of *ADLikelihood*	Difference in Means
1- Negative Framed	286	3.54	3.55	0.004
2- Social Norm	287	3.54	3.64	0.098
3- Positive Framed	301	3.45	3.62	0.179
Overall	874	3.51	3.60	0.095

#### Regression results

Appendix K shows our probit estimation results for wanting more information about ADs after viewing the first framed video (Model 1). The regression contains dummy variables for condition 1 and 2, with condition 3 being the base category. The results are consistent with the findings in Table 5. Those in condition 1, who saw the negative framed video first, were 14 percentage points more likely to want to find out more information about ADs (*p* < .001) compared to those who saw the positive framed video first. The coefficient for those in condition 2, who saw the social norm video first, is positive but not statistically significant. Therefore, compared to those who saw the positive video first, we can’t be sure that those in condition 2 are more likely to want more information about ADs.

Using a *p*-value of .10 or less as a benchmark for statistical significance, several other variables are correlated with wanting more information about ADs. Those who have already spoken to their family about their end-of-life wishes are almost 10 percentage more likely to want more information about ADs compared to those who have not spoken to their family. Those who have had to make decisions about someone else’s end-of-life care are about 7 percentage points more likely to want more information about ADs than someone who has not been involved in these types of decisions for another person.

Most stated reasons for not having an AD were positively and statistically associated with wanting more information after watching the first framed video (not knowing about ADs until now, keep meaning to get around to it, uncomfortable to think about it, process seems complicated, no one has encourage me to get one), with the exceptions of reporting that telling family end-of-life wishes is enough, doctor never discussed ADs, and ‘other’ category. Church goers also wanted more information on end-of-life care, relative to those who do not attend church. In addition, those who believe they have a great deal of choice over their life are significantly more likely to want information about an advance directive. Number of children is positively correlated with wanting more information. However, other demographic variables like race, gender, and age are not statistically significant.

Appendix K also presents the OLS regression results for *ADdiff* (*ADLikelihood* – *SeekLikelihood)* for those who reported not having already sought information on ADs prior to participating in the study. This approximates the change in stated likelihood of obtaining an AD, from after watching the informational video to after watching the first framed video. Since *ADLikelihood* and *SeekLikelihood* are measured on a 1–5 scale, the difference ranges from −4 to 4, with the negative values corresponding to a decrease in likelihood and a value of 4 corresponding to the largest increase in likelihood.

We find that which video was seen first has a statistically significant but small effect on this change. Seeing the negative framed video has a negative effect on *ADdiff*, relative to seeing the positive framed video. Though seeing the social norm video first has a negative coefficient as well, it is not statistically significant. Thus, those who saw the positive framed video on average have a higher value of *ADdiff* compared to those who saw the negative video first, which means a higher approximate change in the stated likelihood of obtaining an AD. This is consistent with the summary statistics in Table 6 above.

A few control variables also statistically affect *ADdiff*. Age has a statistically significant but small impact on the change, with age slightly increasing the stated likelihood of taking action on ADs. Giving the reason of not having an AD because the participant didn’t know about them until now and because no one has encouraged them to get one also have positive and significant effects on the approximate change in stated likelihood of obtaining an AD. On the other hand, having already spoken to family members has a significant and negative impact. Having moderate or conservative political view relative to other views also has a negative impact, as does being more willing to take risks.

The above analysis leads us to the answer to our first research question:

***Result #1:*** Negative message framing about the family burden aspect of end-of-life care statistically increases the choice to obtain more information about advance directives. For those who had not sought information on ADs prior to the study, positive framing has a small positive impact on the approximate change in stated likelihood of obtaining an AD.

### Part one of the study: within participants results

#### Descriptive statistics

Our within participant design results focus on how convincing participants perceived the framed videos to be. After viewing all three of the framed videos, participants were asked to rank the videos in order of how convincing they were (1 being the most convincing and 3 being the least convincing).^6^ Participants ranked the positive framed video as most convincing. Table 7 shows the average ranking of each video.

**Table 7.** Average Ranking of Videos 1- most convincing, 3- least convincing.

Video	Average Rank
Negative Framed	1.98
Positive Framed	1.89
Social Norm	2.13

Examining Table 7 by specific numeric rank, we find the mean rank of the positive framed video to be most convincing. According to a multivariate test of the means, we can reject the null hypothesis that that means are the same at far less than the 1% level (F = 19.39). However, as seen in Table 8, more participants ranked the negative framed video as the most convincing over any of the other videos. The number of participants who ranked the negative framed video as most convincing (432) is statistically significantly higher than the number that ranked the positive framed video (384) and social norm video as most convincing, according to two-sample tests of proportions (344) (*z* = 2.09, *p* = 0.037 and *z* = 3.87, *p* = 0.0001, respectively).

**Table 8.** Ranking of Videos.^8^

Ranking	Negative Framed Video	Positive Framed Video	Social Norm Video
1	432	384	344
2	315	523	322
3	413	253	494

In fact, findings regarding the negative framed video seem to be quite polarizing. Most either ranked it at most effective (37.24%) or least effective (35.6%) as opposed to in the middle (27.16%). We also note many participants ranked the positive framed video as second (45.09%), which increased the positive video’s average rank. So, while on average the positive framed video ranked as more convincing, a larger number of participants ranked the negative framed video first, as the most convincing. Thus, we have the answer to our second research question.

***Result #2***: On average, the positive messaging about the family burden aspect of end-of-life care is perceived as more convincing to obtain an AD, but more participants perceived negative messaging as most convincing over every other video.

#### Regression results

To further discern what significantly affected participant’s ranking of the videos, we estimate an ordered probit regression for the rankings of each of the videos. The results are in Appendix L. Most variables do not have a significant impact on how each video is ranked.

Age does significantly affect ranking of the positive and negative framed videos. For the negative framed video, the positive coefficient on age indicates that as age increases, participants are more likely to rank the negative framed video with a higher number; in other words, older participants rank it as less convincing. For the positive framed video, age has a significant but negative effect. This means that the older a participant is, the more convincing they perceive the positive framed video. Thus, older participants found the positive framed video more convincing than the negative framed video. This result is relevant because the GAO Report (2015) found age to be a significant variable in whether or not someone has an advance directive. Different types of message framing based on age could have differential effects on AD enrollment.

Those who are cohabitating but not married rank the negative framed video as more convincing and the social norm video as less convincing, relative to those who have never married. In addition, the dummy variable indicating if a person has a chronic disease statistically affects how the social norm video is ranked. Chronic disease has a negative coefficient, so a participant is more likely to view the social norm video as more convincing if they have a chronic disease.

***Corollary to Result #2:*** Positive messaging around the family burden aspect of end-of-life care is perceived as more convincing the older participants are, while the negative messaging is perceived as more convincing the younger participants are. Those with a chronic disease find social norm messaging involving someone without an ailment as more convincing, while those who are cohabitating find it less convincing relative to individuals who have never married.

### Part two of the study: following up about AD creation, actions, and intentions

Of the 1,160 participants who participated in part one of the study, 562 answered our questions in part two of the study about one month later, the follow-up survey. Table 9 compares basic demographic descriptive statistics for the sample in each part of the study. There is no significant difference in the demographic variable distributions (race, gender and age) between the two samples.

**Table 9.** Demographic Statistics for Sample in Initial and Follow-Up Survey.

	Part one (N=1160)	Part two (N^#^=561)
Age (mean)	47.57	47.4
Female	75.6%	76.8%
Hispanic or Latino origin	9.7%	9.8%
*Race:*		
White	79.4%	79.1%
Black	13.4%	13.2%
Asian	3.1%	3.6%
American Indian or Alaskan native	0.78%	1.1%
Other	3.4%	3%

^ #^one observation is dropped due to incomplete information.

Of the 562 participants, 106 (18.86%) self-reported they had created an advance directive.^7^ Of the 106 who reported creating an AD, 67 reported using mydirectives.com to create it, the website we suggested that allows for free AD creation. The remaining 39 used an alternative method.

If participants reported they had not created an AD, they were asked if they had talked to anyone about creating their own AD, and if so, to whom they had talked. Of these 455 part two respondents, 113 reported talking to someone about creating and AD. The vast majority, 80% talked to a family member, 9% talked to a medical provider, over 3% to a lawyer, and about 8% to someone else such as a friend.

The 455 participants who reported creating an AD were also asked if they planned to create one. About 14% said no, almost 7% said probably not, 35% said they had not decided, 33% said yes (someday), and almost 11% said yes (soon).

#### Video order

Table 10 shows the percentage in each condition who said they had created an advance directive, said they had talked to someone about creating one, and said they plan to create one someday or soon. For example, 18.1% of those who saw the negative framed video first reported they had created an advance directive.

**Table 10.** Percentage in Each Condition Who Reported Taking Action around ADs.

Condition & First Video Observed	% Reporting Had Created AD (*CreatedAD*=1)	% Reporting Had Spoken to Someone about AD (*SpokenAboutAD*=1)	% Reporting Will Create AD Someday or Soon (*WillCreateAD*=1)
1- Negative Framed	18.1	23.6	40.7
2- Social Norm	18.3	26.9	44.2
3- Positive Framed	20.1	23.9	47.2
Universe	562 Participants in follow-up survey	455 Participants in follow-up survey who had not created AD	455 Participants in follow-up survey who had not created AD

We present this information to explore a possible order effect. Which framed video participants saw first only has a causal interpretation on outcomes measured after the first framed video was seen (such as wanting more information about ADs), not on outcomes measured after all framed videos were seen (such as obtaining an AD). However, since participants were randomly assigned to each condition, if video *order* impacts whether or not someone obtained an AD, that effect can be interpreted causally. In other words, if we find a significant effect of which video was seen first (same as condition) on AD completion, then that must be viewed as an order effect.

There are no statistically significant differences between the three conditions in the percentage who created an AD, according to a Bonferroni multiple-comparison test (F = 0.15, *p* = 0.86). Nor are there differences between the conditions in the percentage who spoke to someone about an AD (F = .28, *p* = .76) or the percentage who said they would create an AD (F = .63, *p* = .53). Therefore, we find the order participants viewed the videos had no effect as to whether participants created an AD. This is not surprising because participants viewed all three videos in a very short amount of time.

#### Ranking of videos

Next we consider how self-reported AD creation is correlated with ranking of the videos in terms of persuasiveness to create an AD. Looking across the rows of Table 11 below, of those who reported they had created an AD, the positive framed video was most often ranked as the most convincing (39%) of the three videos. It was also most often ranked in the middle (46%), and least often ranked least convincing (15%), both by quite a large margin.

**Table 11.** Ranking of Videos by Self-Reported AD Creation in Follow-up Sample.

Ranking*	Negative Framed Video	Positive Framed Video	Social Norm Video
Created AD			
1	29 (27.4%)	41 (38.7%)	36 (34%)
2	28 (26.4%)	49 (46.2%)	29 (27.4%)
3	49 (46.2%)	16 (15.1%)	41 (38.7%)
Total	106 (100%)	106 (100%)	106 (100%)
Did Not Create AD			
1	172 (37.8%)	154 (33.8%)	129 (28.4%)
2	127 (27.9%)	199 (43.7%)	129 (28.4%)
3	156 (34.3%)	102 (22.4%)	197 (43.3%)
Total	455 (100%)	455 (100%)	455 (100%)

*Participant ranking order where 1 is most convincing and 3 is least convincing.

Of those who reported they did not create an AD, the negative framed video was most often ranked as most convincing (38%) by a fairly small margin compared to the positive framed video (34%). Again the positive framed video was most likely to be ranked in the middle in terms of being convincing (44%) and least likely to be ranked least convincing (22%) by quite a large amount.

Looking down the columns of Table 11, a larger percentage of people who reported not creating an AD ranked the negative framed video first or second in terms of persuasiveness than people who reported creating an AD. This is not the case with the other videos. Regarding the positive framed video, the opposite is true; a larger percentage of people who reported creating an AD ranked the positive framed video as first or second compared to those who reported not creating an AD.

Thus, rating the positive framed video as convincing is correlated with reported action to create an AD. Another way to see the trend is in Table 12. Participants who ranked the negative framed video as most convincing were less likely to report creating an AD than those who ranked one of the other two videos as most convincing (14.43% versus 21.03% and 21.69%).

**Table 12.** The Decision to Create an AD by Video Ranked as Most Convincing.

Self-Reported AD Creation (*CreatedAD*)	Video Ranked as Most Convincing (1)		
	Negative Framed Video	Positive Framed Video	Social Norm Video
Yes	29 (14.43%)	41 (21.03%)	36 (21.69%)
No	172 (85.57%)	154 (78.97%)	129 (77.71%)
Sum	201 (100%)	195 (100%)	165 (100%)

#### Information seeking

Not all of the participants who reported creating an AD in the follow-up survey had indicated they wanted more information after watching the first framed video in part one of the study. In fact, 55 of the 106 participants who reported obtaining an advance directive (51.89%) indicated they did not want additional information after the first framed video. Of those who reported creating an AD, 36 had sought information on ADs prior to participating in the study, and 22 had both sought info previously and wanted more information after watching the first framed video.

A possible reason 51.89% of the participants that reported creating an advanced directive indicated they didn’t want additional information about ADs is they were already convinced to obtain one by the time they got to the end of the first framed video and didn’t need to spend the time reading additional information.

We return to a question asked of participants after they viewed their first framed video: ‘How likely are you to ever obtain an advance directive after seeing this video?’ A comparison between likeliness to obtain an AD (*ADLikelihood*) and wanting more information on ADs (*WantInfo*), for those who indicated they created an AD, is shown in Table 13 by condition.

**Table 13.** Comparison of How Likely to Obtain an AD and Wanting More Information after First Framed Video by Condition for Participants Who Reported Creating an AD.

	Condition 1- Negative Frame		Condition 2- Social Norm		Condition 3 – Positive Frame		Total	
How likely to obtain an AD? (*ADLikelihood*)	Wants more info (*WantInfo*=1)	Does not want more info (*WantInfo=0*)	Wants more info (*WantInfo*=1)	Does not want more info (*WantInfo*=0)	Wants more info (*WantInfo*=1)	Does not want more info (*WantInfo*=0)	Wants more info (*WantInfo*=1)	Does not want more info (*WantInfo*=0)
1- Not At All Likely	0	2	0	2	0	4	**0**	**8**
2	0	2	1	1	0	1	**1**	**4**
3	3	5	2	6	3	6	**8**	**17**
4	9	5	6	3	4	6	**19**	**14**
5- Very Likely	4	1	10	4	9	7	**23**	**12**
Total	**16**	**15**	**19**	**16**	**16**	**24**	**51**	**55**

Only Condition 2 participants exhibited a significant difference in the number who indicated they wanted additional information compared to those who didn’t (16 vs 24). They saw the positive framed video first, and recall that at this time had only seen the positive framed video. This is also the only condition in which more participants indicated they did not want more information despite the fact that, of the 24 who said they did not want more information, 19 responded with a 3 or higher that they would be likely to create an advance directive after viewing this video. We’ve already seen that those who reported creating an AD ranked the positive video as most convincing, on average. Hence it is very possible that one reason the majority of the participants who reported creating an AD said they did not want additional information is they had already been convinced to create one. Additional research is needed to draw definitive conclusions.

#### Regression results: what other factors are correlated with AD creation, talking to someone about AD creation, or stating intention to create an AD?

Several characteristics are correlated with self-reported AD creation. Appendix M reports the results of a probit regression with dependent variable *CreatedAD* to investigate factors that may have affected whether an AD was created, for the participants who returned for part two of the study. Ranking the negative framed video as most convincing significantly decreases the likelihood of creating an AD. Thus, those who ranked the positive framed video and social norm video as most convincing are more likely to create an AD. Having sought information about ADs (*SoughtInfo*) prior to participation in part one of the study significantly increases the probability of obtaining an AD, by about 12 percentage points. Those who stated in part one of the study, prior to watching any framed videos, that they did not have an AD because they had made their wishes known to their family are more likely to report having created an AD.

Several demographic variables are statistically correlated with self-reported AD creation. The variable *age* is negative and statistically significant; thus, the older a participant, the less likely they are to have created an advance directive. Females are less likely to report creating an AD. We find race to significantly affect the probability of creating an AD. White participants are more likely to complete an AD, relative to non-white, non-black participants. We also find black participants to be more likely to complete an advance directive than white participants and those of other races.

We also estimate probit models for whether or not participants reported having spoken to someone about ADs since the initial survey, and whether they said they will create an AD either someday or soon (Appendix M). Those who had already sought information about ADs prior to the study, spoken to family members prior to the study, and had been involved in deciding end-of-life care for someone else were more likely to have spoken to someone about ADs between the initial survey and the follow-up survey. Having sought information has the largest impact, increasing the probability of speaking to someone between surveys by over .25 percentage points.

Similarly, having previously sought information and having previously spoken to someone about ADs also significantly affects the likelihood that the participant said they would create an AD someday or soon in the follow-up survey. In addition, wanting for information after the initial video did have a positive impact on reporting AD creation would happen someday or soon, increasing it by about .18 percentage points.

***Result #3***: Those who ranked the positive framed video and social norm video as most convincing were more likely to self-report creating an AD. Several other factors are positively correlated with self-reported AD creation, including being black and white (relative to other races) and choosing ‘my family knows my wishes’ as a reason to not have an AD prior to viewing the framed videos.

## Discussion

We present the results of a survey-based experiment on how message framing on the family burden aspect of end-of-life care is correlated with decisions around advance directives and perceived convincingness to obtain an AD. We find that negative message framing about the family burden aspect of end-of-life care is positively correlated with the choice to obtain more information about advance directives. However, the negative messaging was somewhat polarizing, with more people ranking it as least convincing than ranking it in the middle. For those who had not sought information on ADs prior to the study, positive framing has a small positive impact on the approximate change in stated likelihood of obtaining an AD. On average, positive framing is perceived as more convincing to obtain an AD. Last, ranking the positive framed video as first or second in terms of convincingness is correlated with self-reported creation of an AD, whereas ranking the negative framed video as first or second is correlated with not creating an AD. Social norm messaging is also positively correlated with self-reported obtainment of an AD.

Negative framing may be most effective in encouraging information acquisition as opposed to action, while positive framing may be most effective in encouraging action as opposed to information acquisition. If we view obtaining an AD as preventing against bad circumstances in the future, then this result is consistent with the literature that finds that positive or gain-framed messaging is more effective at promoting prevention behaviors, such as Rothman and Salovey (1997). However, if we view wanting more information as a prevention behavior, then our results are not consistent with previous findings. Wanting more information, however, is not preventive unless the information is consumed at the very least. Taking action to get an AD is a more concrete preventive action. Further analysis into the polarizing nature of the negative messaging may be useful. Knowing the reasons for the polarization could be important to tailor messaging to different demographic groups.

Positive messaging around the family burden aspect of end-of-life care is perceived as more convincing the older participants are, while the negative messaging is perceived as more convincing the younger participants are. Older adults have been found to focus more on positive messages and emotionally positive goals as they age (Carstensen, 2006). This is also supported by findings reported by Sparks and Ledgerwood (2019) that, with aging, negative framing stickiness is attenuated. These results have important implications for message framing about ADs directed to older adults. Additionally, positive framing overall was associated in this study with increased intention to complete an AD regardless of age. Perhaps greater consideration of and emphasis on positive messages should be made more available to the public.

Perceptions of how convincing the types of framing are also depend on whether an individual has a chronic disease, such that those with a chronic disease find social norm messaging involving someone without a chronic ailment as more convincing. This is an interesting result because the social norm video specifically mentions that the third party who passed away had no ailments. It is possible those with ailments find the video that specifies the participant had no ailments as more convincing. More analysis is needed to draw firm conclusions about this issue. In addition, those who are cohabitating but not married rank the negative framed video as more convincing and the social norm video as less convincing, relative to those who have never married. It could be that these participants are more likely to ignore social conventions in various aspects of their lives.

Those who stated in part one of the study, prior to watching any framed videos, that they did not have an AD because making their wishes known to their family was enough were more likely to report having created an AD. Rao et al. (2014) stated this as an established reason people give to not have an advance directive. They do not consider it necessary since they have shared their wishes with their family. Participation in our study likely increased AD completion by individuals listing a common reason to not have one. This finding seems worthy of further inquiry.

Demographic results for participants who requested additional information about ADs and reported obtaining ADs did not generally support current literature. The GAO Report (2015) found that older adults, the more educated, and those with a chronic disease are more likely to have an AD. We did not find those with a chronic disease or higher education to be more likely to complete an advance directive, and we found age to be negatively correlated with AD creation. It may be that many older adults, those with higher education, and those with a chronic disease did not qualify for our study because they indicated that they already had an AD, and the individuals with these demographic characteristics left to participate may be those who do not want an AD for reasons beyond the scope of this study. In addition, since we briefly educated the participants about ADs, general educational attainment may be less relevant in this setting.

We also find that black individuals are more likely to report AD completion, compared to other races. This finding is interesting in conjunction with those of Kwak and Haley (2005), who suggest two reasons for a lack of minority/ethnic enrollment in ADs. The first is a lack of knowledge of advance directives by non-white or ethnic groups. Of the participants identifying as black in our study, 39.4% stated they were not aware of ADs before participating in our study. This is compared to 19.3% of all non-black individuals. Kwak and Haley’s second possible reason for a lack of minority or ethnic group enrollment in ADs is cultural preferences in end-of-life decisions. Our study suggests more education of minority groups could increase their enrollment in ADs, perhaps partially overcoming the cultural preferences. This could be a fruitful area for further research.

This study is exploratory in nature and not without limitations. There is some variation in the length of the videos, which is a potential confounding variable. It is possible a slightly longer video may have more impact on decision making. However, we do find that the shortest video, the positive framed one, is correlated with several positive outcomes, such as having a small positive impact on the approximate change in stated likelihood of obtaining an AD, being ranked as most convincing on average, and being correlated with self-reported AD creation.

In addition, we did not have a condition in which a group of participants watched only the informational video and not any framed videos. Thus, we can’t say that the framed videos alone had a causal impact on the decision to request more information about ADs. Rather, it would be the combination of the informational video and the framing that would have a causal impact. Some participants may have already decided they wanted more information after watching the informational video and before watching the first framed video. However, we do ask participants after the informational video how likely they are to seek information about creating an AD, if they had not already sought information, and are able to leverage this to look at a change in this attitude once they watch the first framed video.

The decision to obtain an advance directive is important to limit the high costs of end-of-life medical care, the emotional and possible financial strain on families, and the possibility that someone does not get the healthcare they desire when they are unable to make their wishes known. This study adds to the sparse literature regarding use of behavioral economics to explore framing effects on decision making around ADs. It has provided several avenues for further research and can inform future studies in a clinical setting. Further studies of behavioral economics and framing can continue to assist policy makers and the medical community in providing the best care possible tailored to the wants and needs of each individual patient.

## Notes

1 Video lengths are slightly different because it takes longer to set up the scene for the negative frame and social norm video compared to the positive framed video, in which there was no conflict or problems to address. To make videos the same length, we would have had to add ‘filler’ material or over emphasize the specific frame whose video needed to be longer. Hence we made a design decision to have the videos parallel each other on the emphasis of the frame, though we realize different lengths of videos is not optimal.

2 The social norm video was added to the study design after pilot data suggested the negative frame was too difficult to watch and, therefore, not convincing. While the participant will of course not know the fictional characters in the video, the video does provide a social norm component utilizing story-telling and the characters agreeing that the enrollment in an advance directive is a good idea. As Courtney et al. (2014) note, this can actually be more powerful testimony than just listing statistics.

3 We acknowledge that every combination of videos was not used. We picked these 3 because each video was first in one condition, second in another condition and third in the final condition. In addition, there is never a case where one frame is viewed before another in all three conditions. As we continue to explore this topic, perhaps other combinations/orders should be tested. Due to budget constraints we were limited in the number of subjects we could survey. Because of this we wanted to be sure we had enough participants per treatment to be able to significantly answer our research questions. As there was no order effect between the three conditions we tested, we do not expect there to be one when the videos are put in any other order. However this does require further testing.

4 Participants were only asked if they wanted more information on advance directives after the first video. This design choice was because we wanted to see which frame on its own was most effective in convincing participants to want to obtain additional information on advance directives. If we had asked the question after the second and third video, it would be impossible to discern the frame (or combination therein) that invoked a positive response.

5 We do not have a group that only sees the informational video, so the true causal effect is a result of the informational video plus the first framed video seen. Everyone sees the same informational video.

6 We did not define ‘convincing’ for the participants, so how a participant ranked the video is based on their own interpretation of what ‘convincing’ is in the context of persuading a participant to create an advance directive.

7 Because of the way the follow-up survey was conducted, we recognize there is a selection bias in the participation of part two surveys. All of those who participated in part one of the survey were invited to participate in part two, until the target number of 562 was reached. Those who completed an advance directive in the month interim period are probably more likely to respond to part 2 of the survey. Hence, it’s possible the advance directive completion percentage out of all of the participants who initially participated to be lower.

8 We tested to see if the ranking of videos was determined by treatment – the order in which they were viewed. We found treatment to be an insignificant variable in determining the ranking of videos.
